# Serious Injury in Metropolitan and Regional Victoria: Exploring Travel to Treatment and Utilisation of Post-Discharge Health Services by Injury Type

**DOI:** 10.3390/ijerph192114063

**Published:** 2022-10-28

**Authors:** Jemma Keeves, Belinda Gabbe, Sarah Arnup, Christina Ekegren, Ben Beck

**Affiliations:** 1 Department of Physiotherapy, Epworth Hospital, Melbourne 3122, Australia; 2 Department of Epidemiology and Preventive Medicine, Monash University, Melbourne 3004, Australia; 3 Rehabilitation, Ageing and Independent Living Unit, Monash University, Melbourne 3004, Australia

**Keywords:** serious injury, traumatic brain injury, orthopaedic injury, spinal cord injury, road trauma, access to healthcare, healthcare utilisation, geography

## Abstract

This study aimed to describe regional variations in service use and distance travelled to post-discharge health services in the first three years following hospital discharge for people with transport-related orthopaedic, brain, and spinal cord injuries. Using linked data from the Victorian State Trauma Registry (VSTR) and Transport Accident Commission (TAC), we identified 1597 people who had sustained transport-related orthopaedic, brain, or spinal cord injuries between 2006 and 2016 that met the study inclusion criteria. The adjusted odds of GP service use for regional participants were 76% higher than for metropolitan participants in the orthopaedic and traumatic brain injury (TBI) groups. People with spinal cord injury (SCI) living in regional areas had 72% lower adjusted odds of accessing mental health, 76% lower adjusted odds of accessing OT services, and 82% lower adjusted odds of accessing physical therapies compared with people living in major cities. People with a TBI living in regional areas on average travelled significantly further to access all post-discharge health services compared with people with TBI in major cities. For visits to medical services, the median trip distance for regional participants was 76.61 km (95%CI: 16.01–132.21) for orthopaedic injuries, 104.05 km (95% CI: 51.55–182.78) for TBI, and 68.70 km (95%CI: 8.34–139.84) for SCI. Disparities in service use and distance travelled to health services exist between metropolitan Melbourne and regional Victoria following serious injury.

## 1. Introduction

Transport-related injuries are expected to become the third leading cause of disability worldwide by 2030 [1]. Despite advances in trauma care, people with orthopaedic injury, traumatic brain injury (TBI), and spinal cord injury (SCI) continue to experience long-term physical disability, psychological dysfunction, and interference from pain [2,3,4]. There is a need to understand whether long-term outcomes for people with serious transport-related injury can be improved through a coordinated and revised approach to post-discharge healthcare.

Urban and regional disparities in access to care exist, with people living in regional areas travelling further to access post-discharge healthcare after major trauma [5]. Both people with serious injury and health professionals have reported limited availability and difficulties accessing necessary care as barriers to health service delivery following injury, particularly for people living in regional areas [6,7,8]. It is unclear whether these barriers to post-discharge care are more significant for people in regional areas as a result of regionalised trauma system design, which centralises higher-level trauma centres in inner metropolitan areas.

Despite survivors of serious injury having long-term and complex healthcare needs, the level of specialised care provided beyond hospital discharge varies depending on the type of injury [4,5,9]. People with TBI and SCI are more likely to receive rehabilitation from specialised services due to the complexity of these injuries [10]. After an orthopaedic injury however, there is no clear pathway for rehabilitation once discharged from a major trauma centre [10]. Given the high prevalence of disability amongst trauma survivors, both with and without serious neurotrauma, consideration for the whole pathway of trauma care from acute management to specialised rehabilitation and community care is pertinent [10].

This novel study is the first to use geospatial analysis to clearly quantify the differences in travel to services and service use for people in different geographic areas by type of injury. The aim of this work was to understand how different injury populations use post-discharge health services across regional and metropolitan areas and explore the distances travelled to health services in the first three years following hospital discharge. Improving our understanding of post-discharge service utilisation is an important step in ensuring necessary services are accessible and available for people with transport-related serious injury.

## 2. Materials and Methods

### 2.1. Study Design

Our registry-based cohort study used linked data from the Victorian State Trauma Registry (VSTR) and Transport Accident Commission (TAC). Our study follows the Strengthening of Reporting of Observational Studies in Epidemiology checklist, see Appendix A [11].

Victoria is the second most populous state of Australia with a population of 6.46 million people, including over 2 million people residing outside the Greater Melbourne region [12]. Victoria has an inclusive trauma system consisting of two adults, and one paediatric, major trauma centres, which are located in metropolitan Melbourne.

The population-based VSTR collects data about all people with major trauma in Victoria, with major trauma defined as: (1) death due to injury; (2) an injury severity score (ISS; based on the abbreviated injury scale (AIS) 2005 version, 2008 update) >12; (3) admission to an intensive care unit >24 h; (4) or an injury requiring urgent surgery [13]. The registry has an opt-out rate <1% and includes data on prehospital care, pre-existing health conditions, injury characteristics and complications, and discharge information [13].

The TAC is Victoria’s no-fault third-party insurer for people who have sustained a transport-related injury, covering medical treatment, rehabilitation, support services, and financial assistance. People are covered by the TAC if their injuries are sustained as a result of driving a car, motorcycle, bus, train, or tram. Cyclists injured in a collision with a moving or stationary motor vehicle (after 9 July 2014) are also covered by the TAC. Pedestrians are covered by the TAC when their injuries arise as a direct result of impact with a motor vehicle, motorcycle, train, or tram. Full details of eligible claimants and expenses covered by the TAC are outlined in the Transport Accident Act 1986 [14]. The TAC collect data pertaining to an individual claim, including detailed information regarding the post-discharge health services paid for by the TAC. These data include details of the date and type of service, service description, and where the service provider is located. The TAC provides these data to the VSTR, linked by claim number. A standardised and secure process is followed to ensure that no patient-level data are provided to the TAC by the VSTR.

### 2.2. Participants

**Figure 1:**
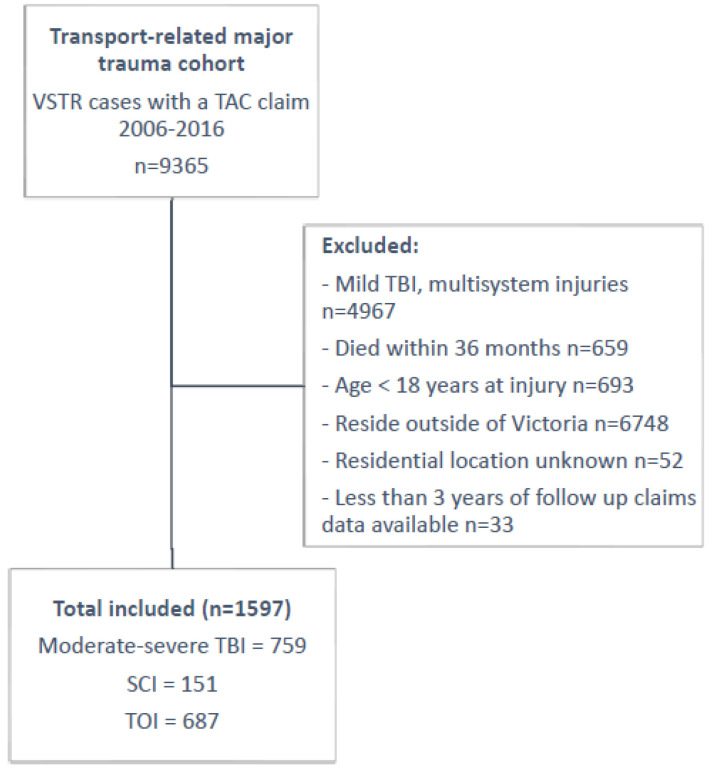
Flow diagram of inclusion criteria.

Victorians who sustained major trauma from a transport-related event between 1 January 2006 and 31 December 2016 with a TAC compensation claim were identified within the VSTR. People were included if they sustained isolated orthopaedic injuries; a moderate to severe TBI or SCI; were aged 18 years or older at the time of injury; had three-years of TAC claims data; and resided in Victoria with a known residential address (Figure 1). The orthopaedic group consisted of people who had sustained an extremity injury with AIS score >1 and/or spine injury with AIS two or three and no other injury with AIS >1 [15]. Traumatic brain injury (TBI) cases were considered to be moderate or severe if they had a head injury with an AIS severity score >2 and the first recorded Glasgow coma scale (GCS) score <13, with or without other system injuries [15]. Mild traumatic brain injuries are not captured by the VSTR unless sustained with other system injures so were excluded from this study. Spinal cord injury was defined as an injury to the spine with an AIS severity score >3, with or without other injuries [15].

### 2.3. Variables

The three key outcomes of interest in this study were: service use, the number of trips per person and distance travelled to health services in the first three years following hospital discharge. Service use was defined as the percentage of participants who used a health service at some point within the study period. The number of trips per person refers to the number of times a health service was visited by service users. Distance travelled was the median trip distance per person from their residential location to the provider location, measured in kilometres.

Health services were categorised as: General Practitioner (GP), other medical professionals (e.g., neurologists, pathologists, psychiatrists, surgeons), mental health services (psychology, social work and case management), physical therapies (physiotherapy, exercise physiology, and hydrotherapy), and occupational therapy (OT). Speech pathology was excluded as this service was used almost exclusively by TBI participants.

### 2.4. Data Measurement

Demographic information, pre-existing health conditions, injury diagnosis and severity, and hospital length of stay and discharge status were extracted from the registry. Data relating to a TAC claim, client address, and service provider locations were provided by the TAC for all services funded between 1 January 2006 and 31 December 2019.

**Figure 2:**
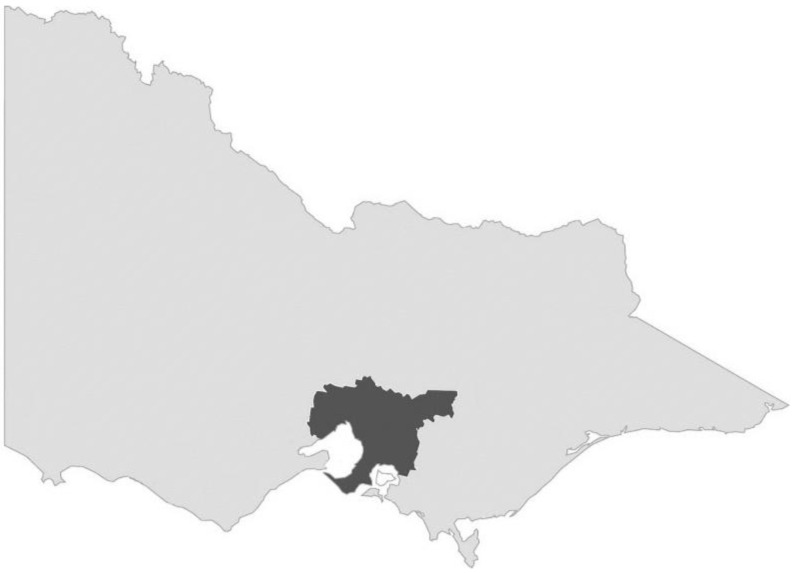
Boundaries for major cities (Greater Melbourne), dark grey, and regional. Victoria, light grey.

Each participant’s residential address at the date of hospital discharge was mapped by their local government area (LGA) to the Accessibility/Remoteness Index of Australia 2016 (ARIA+) and categorised into major city, inner regional, or outer regional [16]. For analysis, the metropolitan group consisted of people living in ‘Major Cities’ and the regional group as people living in ‘Inner Regional’ or ‘Outer Regional’ areas (Figure 2). No participants were residing in ‘Remote’ or ‘Very Remote’ areas by the ARIA+ classification index. Socioeconomic status was categorised using The Index of Relative Socioeconomic Advantage and Disadvantage (IRSAD) according to LGA [17].

Geographic coordinates for each participant’s address and service provider address were compiled through geocoding in RStudio version 3.5.1, and a random sample of 100 were manually checked using Google Maps [18]. Any incomplete provider address locations were entered manually using Google Maps to obtain coordinates. For a single provider it was possible for multiple locations to exist. We mapped the travel distances for all locations using Here Routing API (https://developer.here.com, accessed on 17 Febrary 2020) and used the shortest distance, assuming that people would visit the closest provider location to their homes.

### 2.5. Statistical Methods

Summary statistics were used to describe demographic information, injury characteristics, and service use outcomes. Medians and interquartile range were reported for skewed categorical variables and frequencies and percentages for continuous variables.

Regression models were used to provide estimates of the association between the outcomes of interest and region by injury type. Models were run for each outcome using region as an interaction term with injury type. All models were adjusted for the covariates of age group, sex, Charlson comorbidity index, ISS, and IRSAD based on factors known to impact healthcare utilization [19,20]. Multivariable logistic regression was used for service use (yes/no), and negative binomial regression was used for the number of trips per person, while a general estimating equation (GEE) was used to model distance travelled to services used. For the GEE, a Gaussian model and identity link was used, and an exchangeable correlation was assumed between trips to the same service within each individual. Adjusted odds ratios (OR) and incidence rate ratios (IRR), and the corresponding 95% confidence intervals were calculated for the logistic and negative binomial regression models, respectively. As distance travelled was positively skewed, the data were log transformed before modelling. Model fit was evaluated for concordance and discrimination using residual plots [21]. All analyses were completed in Stata Version 16.0 with the exception of the geospatial analyses which were conducted using RStudio version 3.5.1 [18].

## 3. Results

There were 9365 cases of transport-related major trauma identified from the VSTR; 17.1% (*n* = 1597) were eligible for this study (Figure 1). The characteristics of included participants for each injury group are presented in Table 1.

Across all injury groups, most participants were men and the median age was 33 years (IQR 23–48). Thirty-five percent of participants resided in regional areas. Most participants were injured in motor vehicle or motorcycle crashes. The SCI group had the longest length of acute hospital stay. In the first three years following hospital discharge, the 1597 participants visited health services 159,090 times for GP services, other medical appointments, mental health services, physical therapies, and OT (Table 2).

**Figure 3:**
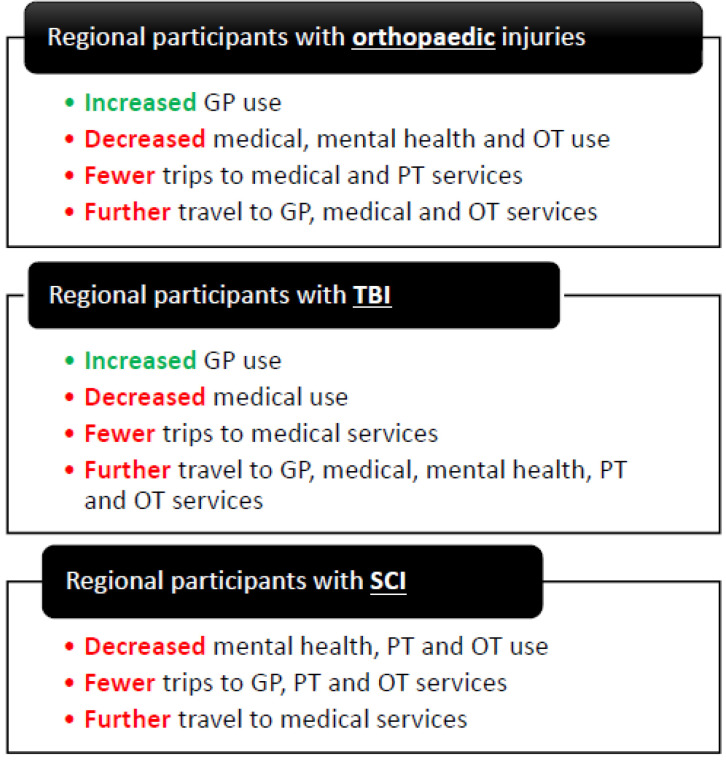
Summary of key findings for regional participants compared with participants in major cities.

Figure 3 provides a summary of the key findings from the multivariable regression analysis for service use, number of trips, and distances travelled to services. More specific results from the models for each outcome and injury group are reported in each section below.

### 3.1. Service Use

**Figure 4:**
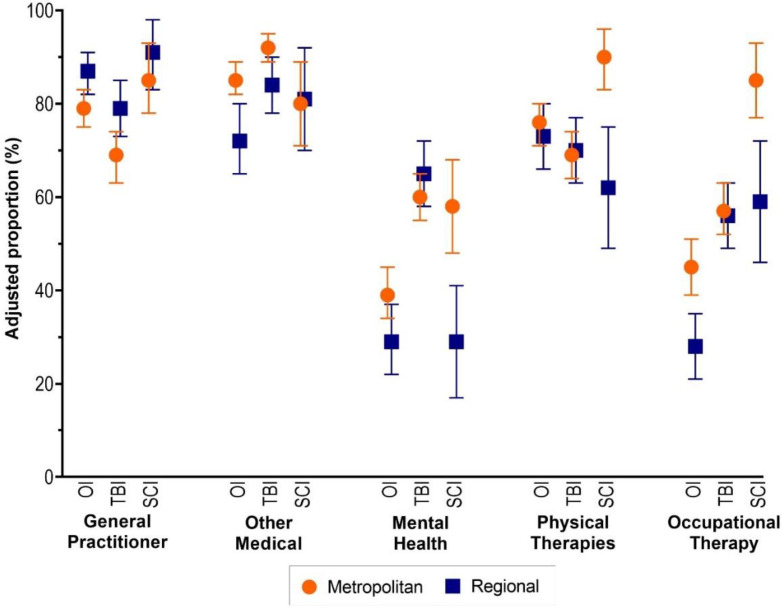
Adjusted proportion of service use by injury group and region.

The adjusted proportions of people using GP services were higher for regional participants in all injury groups (Figure 4). Across all other services, the adjusted proportions for service use were greater for people living in major cities compared with people living in regional areas, except for people with TBI accessing mental health services.

In the orthopaedic and TBI groups, participants in regional areas, compared with major cities, had 76% higher adjusted odds of seeing a GP but 56% and 57% lower adjusted odds of attending other types of medical services, respectively (Table 3). In the orthopaedic group, participants in regional areas, compared with major cities, had 37% lower adjusted odds of attending mental health services and 45% lower adjusted odds of attending occupational therapy services. In the SCI group, participants in regional areas, compared with major cities, had 72% lower odds of accessing mental health, 82% lower adjusted odds of accessing physical therapies, and 76% lower adjusted odds of accessing OT services (Table 3).

### 3.2. Number of Trips

**Figure 5:**
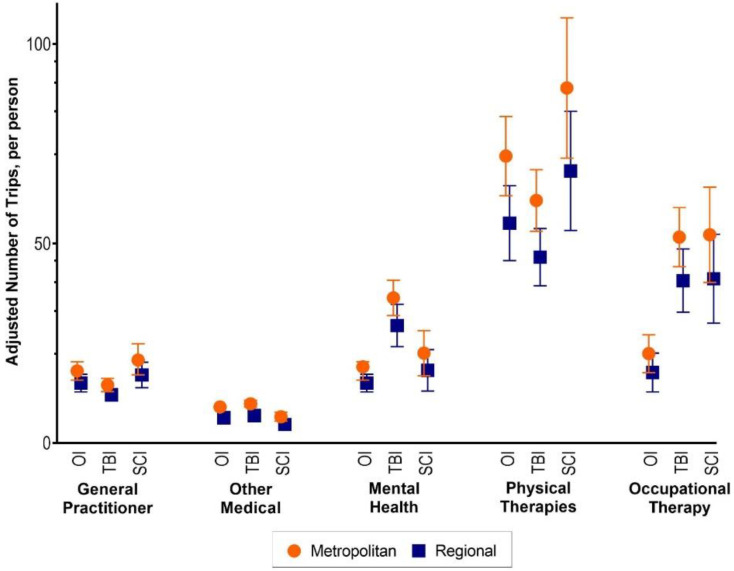
Adjusted number of trips to services in the first three years post-discharge by service type and injury group.

For all injuries and service types, people in regional areas used fewer services than people residing in major cities after adjusting for covariates (Figure 5). Physical therapies were the most commonly used service across all injury groups. In the orthopaedic group, the mean number of trips for participants in regional areas, compared with major cities, was 29% lower for medical services and 24% lower for physical therapy services. In the TBI group, the mean number of trips for participants in regional areas, compared with major cities, was 35% lower for medical services. In the SCI group, the mean number of trips for participants in regional areas, compared with major cities, was 35% lower for GPs, 37% lower for physical therapy and 45% lower for OT services (Table 3).

### 3.3. Distance Travelled

**Figure 6:**
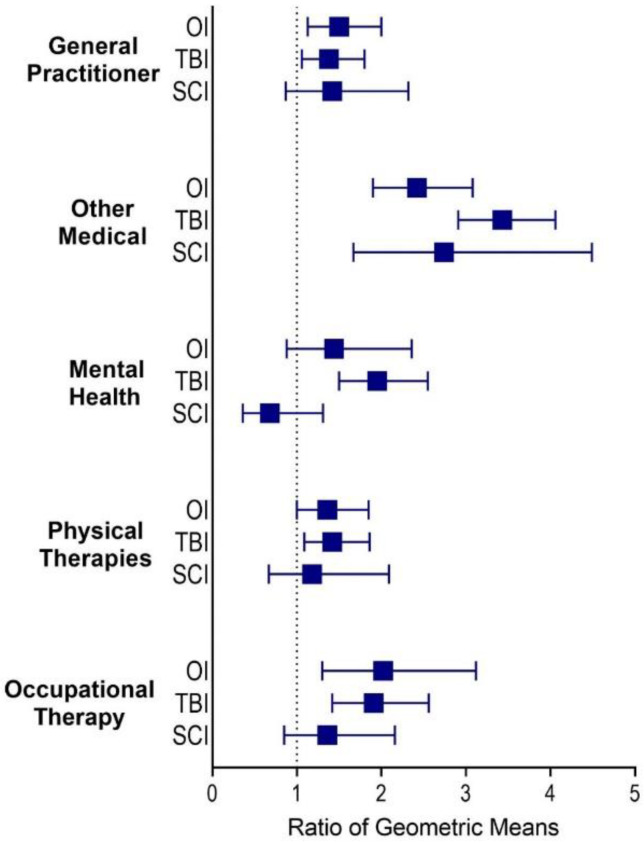
Ratio of geometric means for distance travelled by people in regional areas compared with major cities by injury group and service type.

In the TBI group, participants in regional areas travelled significantly further to access all post-discharge health services compared with participants in major cities (Figure 6). In the SCI group, however, participants in regional areas travelled further only to attend medical services (RGM 2.66, 95%CI 1.63–4.36) (Figure 6). In the orthopaedic group, participants in regional areas travelled 1.4 times further to see a GP (95%CI 1.06–1.88), 2.26 times further to attend other medical services (95%CI 1.76–2.89), and 1.7 times further to OT services (95%CI 1.06–2.62) compared with participants in major cities.

**Figure 7:**
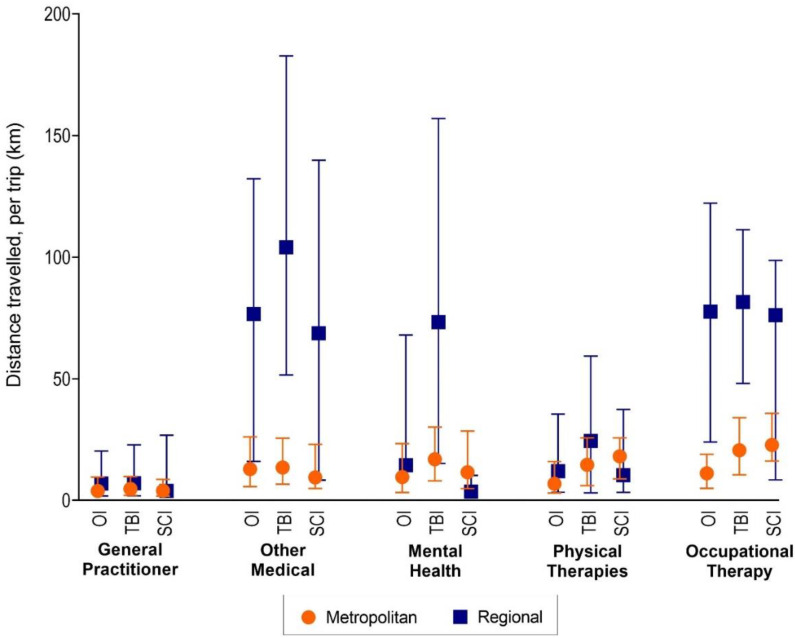
Median and IQR of raw distances travelled to healthcare by service type and injury group.

For visits to medical services, the median trip distances for participants in regional areas with any injury type ranged from 68.70 km (95%CI: 8.34–139.84) to 104.05 km (95% CI: 51.55–182.78) (Figure 7). Comparatively, the median trip distances for participants in major cities with any injury type ranged from 9.44 km (95%CI 4.92–23.05) to 13.50 km (95% CI 6.65–25.59) (Figure 7).

## 4. Discussion

In this study, we compared health service usage and distances travelled by people with transport-related orthopaedic, brain and spinal cord injuries across regional and metropolitan Victoria in the first three years following hospital discharge. For most services and injury types, people in regional areas used fewer services but travelled further to access them than people in metropolitan areas. People with orthopaedic injuries and TBI in regional areas had greater odds of seeing a GP compared with their metropolitan counterparts. This research provides an important contribution to our understanding of how geography impacts healthcare utilisation following major trauma.

We found that regional participants with orthopaedic injuries and TBI had greater odds of attending GP services than metropolitan participants, despite having to travel further. This may be explained by people in metropolitan areas living closer to trauma centres with better access to specialised rehabilitation providers, therefore being less reliant on their local GPs [23,24,25]. Following major trauma, GPs play a critical role in providing ongoing community support, monitoring for secondary complications of injury and psychosocial issues, and assisting in the patient’s return to work [26]. For people living in metropolitan areas, it is possible that these issues may be monitored by a specialised rehabilitation team, including allied health and specialist physicians. Our findings highlight the importance of regional-based GPs having adequate knowledge of injury complications and a network of specialists that may be able to carry out shared virtual consultations to ensure timely and effective management closer to home [27].

Consistent with previous research, our findings suggest that people with serious injuries living in regional areas use fewer health services than their metropolitan counterparts [28,29,30,31]. Having to travel further to access healthcare for people in regional areas may limit accessibility [5,8,24]. Compounding the challenge of distance, transportation difficulties [6,8,29,32] and a limited availability of skilled providers [7,33] have been reported as barriers to accessing necessary services for people with orthopaedic injuries, TBI, and SCI, particularly for those in regional areas. A key consequence of reduced service use is that people with serious injuries living in regional areas often report higher levels of unmet care needs [25,30,34,35,36]. Ensuring the availability of local infrastructure or alternate service delivery methods is essential for people with serious injury due to the chronicity of the condition [8,34,37].

In this study, we found that for all injury types, people living in regional areas travelled further than people in metropolitan areas to access all services. However, after adjusting for covariates, our findings were more nuanced. People living in regional areas with TBI travelled significantly further to all health services than those in metropolitan areas, whereas for people with SCI, a significant difference was only found for travel to medical services, which was based on region. Due to the complexity and long-term issues associated with SCI, people with SCI may choose to live in areas where they can access necessary services [24]. In comparison, given the varying degree of severity of TBI, some people with TBI may place less importance on ease of service access and availability when deciding where they want to reside. These novel findings reinforce the importance of specialised telehealth services and outreach clinics for people in regional areas with TBI to reduce travel burden and ensure access to adequately skilled healthcare services.

In addition to considering alternate service delivery modes, at a systems level, this research contributes a new perspective for post-discharge care coordination for people with serious injury. Policy makers involved in the planning of healthcare pathways across the continuum of care should consider the extra distances travelled by people in regional areas and possible travel burden, which may impact post-discharge healthcare utilisation. Further research to understand patterns of health service utilisation for other groups at risk of health inequities, such as older adults, Aboriginal and Torres Strait Islander People, and culturally and linguistically diverse communities, particularly in regional areas, will further aid in improving healthcare planning.

### Study Limitations

This population-based cohort study provides novel insights into geographic variations in healthcare use following transport-related orthopaedic, brain, and spinal cord injury. However, a limitation of this work was that due to multiple service provider locations being provided, we assumed that an individual attended the closest facility to their home and used the shortest trip distance. This study also only included services that were centre-based; therefore, for people with TBI and SCI, who are likely to have received services in the community or at home, the number of services used may be underrepresented. This also includes care from the Spinal Community Integration Service, a Victorian program that provides people with SCI assistance with returning home and participating in their communities in the first 12 months following discharge. Due to the nature of how these services are billed to the TAC, it was not possible to ascertain specific details of what services were provided on exact dates and at specific locations. However, as this was the same for both regional and metropolitan participants, this is unlikely to have impacted the regional variation within groups. It was also assumed that participants all travelled by car to attend services. Due to the reimbursement available for taxi travel and motorised travel expenses for TAC patients, it is most likely that participants would choose one of these options over human-powered or public transport.

## 5. Conclusions

Health service use following traumatic orthopaedic, brain, and spinal cord injury is complex and continues for years following the initial injury. This research has identified disparities in service use and distances travelled to health services across metropolitan and regional Victoria following serious injury. With people in regional areas using fewer services, except for GPs, and attending these services less often, there is a risk of unmet service needs for these individuals. An increased travel distance to services is one factor that may be contributing to the inequality in access to healthcare in regional areas compared with metropolitan areas. These findings reinforce the need for a review of how specialised rehabilitation services are delivered to people residing in regional areas following major trauma and whether access to post-discharge services is available to everyone long-term, regardless of where they reside. Further research exploring whether there is an association between service use, distance travelled, and health outcomes is necessary to ensure post-discharge care is optimised for people with serious injuries.

## Figures and Tables

**Table 1:** Demographics and injury characteristics of participants.

	All Cases (1597)	TOI (687)	TBI (759)	SCI (151)
	*n* (%)	*n* (%)	*n* (%)	*n* (%)
**Gender**				
Male	1122 (70.3)	461 (67.1)	537 (70.8)	124 (82.1)
Female	475 (29.7)	226 (32.9)	222 (29.2)	27 (17.9)
**Age group, years**				
18–24	480 (30.1)	153 (22.1)	297 (39.1)	31 (20.5)
25–34	367 (23.0)	151 (22.0)	175 (23.1)	41 (27.2)
35–44	275 (17.2)	123 (17.9)	123 (16.2)	29 (19.2)
45–54	206 (12.9)	104 (15.1)	82 (10.8)	20 (13.2)
55–64	120 (7.5)	67 (9.8)	42 (5.5)	11 (7.3)
65–74	76 (4.8)	39 (5.7)	24 (3.1)	13 (8.6)
75+	73 (4.6)	51 (7.4)	16 (2.1)	6 (4.0)
**Injury Severity Score, median (IQR)**	20 (13–29)	13 (9–14)	29 (22–38)	29 (24–33)
**CCI [22] weight (CCI) [1]**				
0	857 (53.5)	521 (75.6)	235 (30.8)	101 (66.4)
1	582 (36.4)	126 (18.3)	424 (55.6)	33 (21.7)
>1	142 (8.9)	32 (4.6)	98 (12.9)	13 (8.6)
**Acute hospital LOS (days), median (IQR)**	13 (7–24)	8 (5–13)	18 (11–29)	24 (13–39)
**Region (ARIA+ 2016)**				
Major cities	1040 (65.1)	441 (65.2)	507 (66.8)	92 (60.9)
Inner regional	438 (27.4)	184 (26.8)	205 (27.0)	49 (32.5)
Outer regional	119 (7.5)	62 (9.0)	47 (6.2)	10 (6.6)
**Socioeconomic status (IRSAD)**				
1 (most disadvantaged)	268 (16.8)	128 (18.6)	118 (15.5)	22 (14.5)
2	168 (10.5)	72 (10.5)	81 (10.6)	15 (9.9)
3	309 (19.3)	128 (18.6)	150 (19.8)	31 (20.5)
4	432 (27.1)	189 (27.5)	202 (26.6)	41 (27.2)
5 (least disadvantaged)	420 (26.3)	170 (24.7)	208 (27.4)	42 (27.8)
**Discharge destination**				
Home	287 (18.0)	237 (34.5)	41 (5.4)	9 (6.0)
Other (e.g., inpatient rehabilitation)	1310 (82.0)	450 (65.4)	718 (94.6)	142 (94.0)
**Road user [2]**				
Motor vehicle driver or passenger	881 (55.2)	348 (50.6)	451 (59.5)	82 (53.9)
Motorcyclist	347 (21.7)	198 (28.8)	99 (13.0)	50 (32.9)
Pedestrian	244 (15.4)	85 (12.4)	154 (20.3)	6 (4.0)
Bicyclist	91 (5.7)	42 (6.1)	41 (5.4)	8 (5.3)

*n* = 19 missing [1]. *n* = 34 other or missing [2]. LOS = length of stay; IQR = interquartile range; CCI = Charlson comorbidity index, ARIA+ = Accessibility/Remoteness Index of Australia; IRSAD = Index of Relative Socio-economic Advantage and Disadvantage.

**Table 2:** All services used by participants in the first three years post-discharge by injury type.

	All Cases		TBI		SCI		TOI	
	(*n* = 1597)		(*n* = 759)		(*n* = 151)		(*n* = 687)	
	*n*	%	*n*	%	*n*	%	*n*	%
Physiotherapy	59,532	30.7	27,556	27.0	9667	31.7	22,309	36.5
Occupational Therapy	34,268	17.7	23,026	22.5	6557	21.5	4685	7.7
GP Consult	20,078	10.4	8791	8.6	2937	9.6	8350	13.7
Psychology	18,907	9.7	14,480	14.1	1285	4.2	3142	5.1
Nursing	13,289	6.9	2436	2.4	6032	19.8	4821	7.9
Medical (other)	10,972	5.7	6315	6.2	803	2.6	3854	6.3
Speech Therapy	8373	4.3	8143	7.9	165	0.5	65	0.1
Hydrotherapy	6917	3.6	1727	1.7	233	0.8	4957	8.1
Exercise Physiology	6832	3.5	1816	1.8	839	2.8	4177	6.8
Vocational counselling	4175	2.2	1720	1.7	359	1.2	2096	3.4
Social Work	1792	0.9	1203	1.2	313	1.0	276	0.5
Psychiatry	1584	0.8	1049	1.0	80	0.3	455	0.7
Podiatry	1193	0.6	348	0.3	551	1.8	294	0.5
Osteopathy	1017	0.5	306	0.3	112	0.4	599	1.0
Dental	990	0.5	864	0.8	55	0.2	71	0.1
Dietitian	800	0.4	511	0.5	186	0.6	103	0.2
Case Conferences	741	0.4	444	0.4	30	0.1	267	0.4
Chiropractor	680	0.4	380	0.4	66	0.2	234	0.4
Attendant carer	623	0.3	444	0.4	119	0.4	60	0.1
Paramedical (other)	557	0.3	443	0.4	36	0.1	78	0.1
Acupuncture	468	0.2	275	0.3	29	0.1	164	0.3
Optical	291	0.2	257	0.3	6	<0.1	28	<0.1
Total	194,079		102,534		30,460		61,085	

**Table 3:** Regional variation in service use and number of trips per person in the first three years following hospital discharge determined by multivariable regression analysis.

	Participants Using Service (*n*, %)	Service Use, Adjusted OR (95%CI)	*p* *	Trips per Person, Median (IQR)	Adjusted IRR (95%CI)	*p* ^†^
**General Practitioner**						
TOI						
Major cities	329 (74.6)	Reference		9 (4–22)	Reference	
Regional	206 (83.7)	1.76 (1.1–2.9)	**0.02**	9 (3–21)	0.81 (0.66–0.99)	**0.04**
TBI						
Major cities	370 (73.0)	Reference		9 (3–18)	Reference	
Regional	211 (83.7)	1.76 (1.1–2.8)	**0.02**	10 (4–22)	0.90 (0.75–1.1)	0.31
SCI						
Major cities	80 (87.0)	Reference		18.5 (10.5–30)	Reference	
Regional	54 (91.5)	1.73 (0.5–5.4)	0.35	15 (6–23)	0.65 (0.5–0.9)	**0.02**
**Medical Specialists**						
TOI						
Major cities	360 (81.6)	Reference		5 (2–11)	Reference	
Regional	160 (65.0)	0.44 (0.28–0.70)	**0.001**	3 (2–8)	0.71 (0.6–0.9)	**<0.001**
TBI						
Major cities	477 (94.1)	Reference		8 (4–14)	Reference	
Regional	220 (87.3)	0.43 (0.24–0.78)	**0.01**	5 (3–9.5)	0.65 (0.6–0.8)	**<0.001**
SCI						
Major cities	76 (82.6)	Reference		4 (2–9.5)	Reference	
Regional	49 (83.1)	1.07 (0.42–2.70)	0.89	4 (2–8)	0.90 (0.6–1.2)	0.51
**Mental Health**						
TOI						
Major cities	158 (35.8)	Reference		11 (4–26)	Reference	
Regional	56 (22.8)	0.63 (0.41–0.96)	**0.03**	7 (4–23)	0.88 (0.6–1.3)	0.47
TBI						
Major cities	330 (65.1)	Reference		23.5 (8–54)	Reference	
Regional	169 (67.1)	1.23 (0.84–1.81)	0.29	16 (6–36)	0.78 (0.6–1.0)	**0.05**
SCI						
Major cities	57 (62.0)	Reference		14 (7–35)	Reference	
Regional	17 (28.8)	0.28 (0.1–0.6)	**0.001**	10 (4–17)	0.84 (0.5–1.5)	0.58
**Physical Therapies**						
TOI						
Major cities	322 (73.0)	Reference		42 (15–102)	Reference	
Regional	176 (71.5)	0.87 (0.57–1.33)	0.54	34 (14–74.5)	0.76 (0.6–0.9)	**0.01**
TBI						
Major cities	355 (70.0)	Reference		40 (14–85)	Reference	
Regional	181 (71.8)	1.08 (0.72–1.62)	0.71	34 (10–72)	0.81 (0.6–1.0)	0.07
SCI						
Major cities	84 (91.3)	Reference		87 (41–131.5)	Reference	
Regional	39 (66.1)	0.18 (0.07–0.45)	**<0.001**	46 (9–92)	0.63 (0.41–0.95)	**0.03**
**Occupational Therapy**						
TOI						
Major cities	165 (37.4)	Reference		9 (2–25)	Reference	
Regional	55 (22.4)	0.45 (0.29–0.69)	**<0.001**	4 (2–22)	0.76 (0.5–1.1)	0.17
TBI						
Major cities	317 (62.5)	Reference		30 (8–73)	Reference	
Regional	154 (61.1)	0.95 (0.65–1.39)	0.81	24.5 (5–57)	0.89 (0.7–1.2)	0.40
SCI						
Major cities	81 (88.0)	Reference		49 (14–93)	Reference	
Regional	39 (66.1)	0.24 (0.10–0.58)	**0.001**	11 (3–62)	0.55 (0.3–0.9)	**0.01**

* *p* value for the logistic regression analysis of service use. ^†^ *p* value for negative binomial regression analysis of number of trips, per person, for participants who used that service. *p* values in bold type are significant. IQR = interquartile range, OR = odds ratio, IRR = incidence rate ratio.

## Data Availability

Not applicable.

## Appendix A

**Table A1:** Strengthening The Reporting of Observational Studies in Epidemiology (STROBE) statement checklist.

		Recommendation	Page
**Title and abstract**	1	(*a*) Indicate the study’s design with a commonly used term in the title or the abstract	1
		(*b*) Provide in the abstract an informative and balanced summary of what was performed and what was found	1
**Introduction**			
Background/rationale	2	Explain the scientific background and rationale for the investigation being reported	1
Objectives	3	State specific objectives, including any prespecified hypotheses	2
**Methods**			
Study design	4	Present key elements of study design early in the paper	2
Setting	5	Describe the setting, locations, and relevant dates, including periods of recruitment, exposure, follow-up, and data collection	2–3
Participants	6	(*a*) Give the eligibility criteria and the sources and methods of selection of participants. Describe methods of follow-up	3–4
		(*b*) For matched studies, give matching criteria and number of exposed and unexposed	n/a
Variables	7	Clearly define all outcomes, exposures, predictors, potential confounders, and effect modifiers. Give diagnostic criteria, if applicable	4
Data sources/measurement	8	For each variable of interest, give sources of data and details of methods of assessment (measurement). Describe comparability of assessment methods if there is more than one group	2–3
Bias	9	Describe any efforts to address potential sources of bias	-
Study size	10	Explain how the study size was arrived at	Figure 1
Quantitative variables	11	Explain how quantitative variables were handled in the analyses. If applicable, describe which groupings were chosen and why	4
Statistical methods	12	(*a*) Describe all statistical methods, including those used to control for confounding	4
		(*b*) Describe any methods used to examine subgroups and interactions	4–5
		(*c*) Explain how missing data were addressed	n/a
		(*d*) If applicable, explain how loss to follow-up was addressed	n/a
		(*e*) Describe any sensitivity analyses	-
**Results**			
Participants	13	(a) Report numbers of individuals at each stage of study, e.g., numbers potentially eligible, examined for eligibility, confirmed eligible, included in the study, completing follow-up, and analysed	5
		(b) Give reasons for non-participation at each stage	n/a
		© Consider use of a flow diagram	Figure 1
Descriptive data	14	(a) Give characteristics of study participants (e.g., demographic, clinical, social) and information on exposures and potential confounders	Table 1
		(b) Indicate number of participants with missing data for each variable of interest	n/a
		(c) Summarise follow-up time (e.g., average and total amount)	5
Outcome data	15	Report numbers of outcome events or summary measures over time	5–8
